# Predictive Regression Equations of Flowmetric and Spirometric Peak Expiratory Flow in Healthy Moroccan Adults

**DOI:** 10.1155/2017/8985067

**Published:** 2017-03-30

**Authors:** Khalid Bouti, Iliass Maouni, Jouda Benamor, Jamal Eddine Bourkadi

**Affiliations:** ^1^Centre d'Etudes Doctorales des Sciences de la Vie et de la Santé (CEDOC SVS), Faculty of Medicine and Pharmacy, UM5, Rabat, Morocco; ^2^Center of Tuberculosis and Lung Diseases, SRES, Tetouan, Morocco; ^3^Regional Hospital Center, Tetouan, Morocco; ^4^Faculty of Medicine and Pharmacy, UM5, Rabat, Morocco; ^5^Lung Function Department, Moulay Youssef University Hospital, Rabat, Morocco; ^6^Pulmonology Department, Moulay Youssef University Hospital, Rabat, Morocco

## Abstract

*Introduction*. PEF has never been characterized among healthy Moroccan adults. The objective of this study is to describe the values of PEF among healthy Moroccan adults, to study its relationship with anthropometric parameters (gender, age, height, and weight), to compare spirometric and flowmetric PEF, to establish the prediction equations for PEF, and to study the correlation between PEF and FEV1.* Methods*. Cross-sectional study conducted between May and June 2016. It involved healthy nonsmoking volunteers living in Tetouan, Morocco, gathered through a mobile stand realization of spirometry and peak flow measurements.* Results*. Our final sample concerned 313 adults (143 men and 170 women). For both men and women, age and height were the main determinants of PEF, and a positive correlation was found between PEF and FEV1.* Conclusion*. Our study has established the PEF predictive equations in the Moroccan adult population. Our results allow us to conclude that the PEF can be a reliable alternative of FEV1 in centers not equipped with spirometry.

## 1. Introduction

Peak Expiratory Flow (PEF) is the maximum flow generated during forced expiration following a forced inspiration reaching total lung capacity. This setting is useful for diagnosis of asthma if diurnal variability is greater than 20% for at least three days in a week for two weeks, for its follow-up, and for the evaluation of an asthmatic patient in time of his crisis to decide his hospitalization or his output. It is measured by peak flow meter, which is simple to make, easy to read, less expensive, and available in general medicine. Despite the accuracy of the spirometer, it is more expensive and less available out of pulmonology offices, and its interpretation requires previous learning.

PEF depends on sex, age, height, weight, and ethnicity. In 1963, Leiner et al. established two PEF prediction equations based on age and height for males and females among the American population using a Wright peak flow meter [1]. Nunn and Gregg used the same material to establish similar equations in England, also depending on age and height [2, 3].

No previous study focused on the PEF in the Moroccan population.

The objectives of this study are

- to characterize the values of the PEF in healthy nonsmoking Moroccan adults;
- to study the relationship between PEF and the anthropometric parameters (sex, age, height, and weight).
- to compare spirometric and flowmetric PEF;
- to establish predictive equations of PEF;
- to compare PEF and Forced Expiratory Volume in 1 second (FEV1) in this sample.

## 2. Methods

We have conducted a cross-sectional study for 2 months (May and June 2016).

Our study took the form of a mobile stand for measuring lung function in the following: a public facility (3 days), a higher Institute (3 days), and the Center of diagnosis of tuberculosis and lung diseases (5 days), all in the city of Tetouan, in the north of Morocco.

The stand was of open access for any volunteer wishing to measure his respiratory function. Only the volunteers apparently healthy and giving their consent to participate in the study have been included in it. The selection was based on inclusion and exclusion criteria.

The inclusion criteria were to validate our questionnaire, to perform correctly spirometry and flowmetry maneuvers, to give consent to participate to this study, and to hold Moroccan nationality (participant and his parents).

Exclusion criteria were the presence of any type of respiratory, Ear-Nose-Throat (ENT), heart, osteoarticular, neurological, or hematological affection; the presence of a smoking history; a high-level sport practice; a Body Mass Index (BMI) ≥ 30 Kg/m^2^; pregnancy; or spirometric abnormal curve.

Height and weight data were noted using a wooden wall growth height chart (Dancing Mole, Salisbury, United Kingdom) and a mechanical weighing scale (Zhongshan Camry Electronic, Zhongshan, Guangdong, China).

The realization of the study required the mobilization of 2 portable spirometers type SPIROLAB III (Medical International Research, Roma, Italy) meeting the criteria of American Thoracic Society/European Respiratory Society (ATS/ERS) as well as of peak flow meters type Mini-Wright (Clement Clarke International Ltd., London, UK) [4]. SPIROLAB III calibration is not compulsory, but we made tests with a calibrated 3-litre calibration syringe every morning. All the Mini-Wright peak flow meters were new, so they did not require any calibration or stability verification.

*Flowmetric Peak Expiratory Flow* (F-PEF) has been measured three times for each participant; the best performance was chosen and graded.

The measurement of* the Spirometric Peak Expiratory Flow* (S-PEF) and FEV1 required 3 to 5 spirometric forced maneuvers respecting the acceptability and reproducibility criteria cited in the ATS/ERS recommendations [5], and we noted the best values.

All results were transferred and processed by the Statistical Package for the Social Sciences (SPSS) software (version 22.0). Correlations between each anthropometric parameter (sex, age, height, and weight) and the F-PEF, S-PEF, and FEV1 were studied in a bivariate way using the Pearson correlation coefficient. The relationship between the parameters, with a positive correlation between the F-PEF, S-PEF, and the FEV1, has been studied in simple linear regression. The parameters that have a significant correlation in simple linear regression were analyzed in multiple linear regression to retain the influential parameters for F-PEF, S-PEF, and FEV1 in a statistically significant way.

In the end, we could establish prediction equations of F-PEF, S-PEF, and FEV1 for each gender based on anthropometric parameters (age, weight, and height).

The correlation between the parameters of this respiratory work (F-PEF, S-PEF, and FEV1) was studied two by two using Pearson correlation coefficient.

In case of positive correlation, each two parameters have been studied in simple linear regression. Then, these parameters prediction equations were established.

This study is part of the GLIM project (Global Lung Initiative in Morocco) which received the approval of the Committee of Ethics of the Faculty of Medicine and Pharmacy of Rabat.

## 3. Results

### 3.1. Descriptive Study

After application of inclusions and exclusions criteria on the 405 volunteers who came to our stand, 92 subjects were excluded: 42 subjects on questionnaire, 5 for foreign nationalities, and 45 for bad maneuvers. In the end, only 313 volunteers were selected; 143 men (45.68%) and 170 women (54.31%), aged between 18 and 70 years.

Table 1 summarizes the anthropometric and ventilatory characteristics (F-PEF, S-PEF, and FEV1) of the entire sample and of men and women separately. The mean S-PEF for the entire sample is greater than the F-PEF (*p* < 0.0001). We also note that the average value of the S-PEF is superior to the F-DEP among both sexes, with, respectively, 527 ± 113 l/min versus 460 ± 127 l/min for men and 360 ± 75 l/min versus 303 ± 81 l/min for women (*p* < 0.0001).

### 3.2. Analytic Study

#### 3.2.1. The Relationship between F-PEF and Height, Age, and Weight

Table 2 shows the correlation of the F-PEF with age, height, and weight in simple and multiple linear regressions in the 2 sexes. We can deduce through this analysis the predictive equations of F-PEF from age and height among males: F-PEF = −374 − 1.63 Age (years) + 5.20 Height (cm) and among females: F-PEF = −235 − 0.24 Age (years) + 3.48 Height (cm).

#### 3.2.2. The Relationship between S-PEF and Age, Height, and Weight

Table 3 shows the correlation of the S-PEF with age, height, and weight in simple and multiple linear regressions among the 2 sexes. We can deduce through this analysis the predictive equations of PEFs from age, height, and weight among males: S-PEF = −50 − 2.11 Age (year) + 2.61 Height (cm) + 2.75 weight (Kg) and from the age and height among females: S-PEF = −241 − 0.61 Age (year) + 3.79 Height (cm).

#### 3.2.3. The Relationship between FEV1 and Age, Height and Weight

Table 4 shows the correlation of decline with age, height, and weight in simple and multiple linear regressions in the 2 sexes. We can then deduce predictive equations among males from the age and the height of this analysis: FEV1 = −3.66 − 0.03 Age (year) + 0.05 Height (cm) and among females from the age and height: FEV1 = −2.83 + −0.02 Age (year) + 0.04 Height (cm).

#### 3.2.4. Correlation between F-PEF and S-PEF

Among males, we found a strong positive correlation between F-PEF and S-PEF (*r* = 0.73; *p* < 0.0001). Among females, we found a strong positive correlation between F-PEF and S-PEF (*r* = 0.72; *p* < 0.0001). We can then deduce from this analysis the predictive equations of S-PEF from F-PEF among males: F-PEF = 25 + 0.82 S-PEF and females: F-PEF= 21 + 0.78 S-PEF.

#### 3.2.5. Correlation between F-PEF and FEV1

Among males we found a positive correlation between F-PEF and FEV1 (*r* = 0.43; *p* = 0.005). Among the female sex, we found a positive correlation between the F-PEF and FEV1 (*r* = 0.25; *p* = 0.006). We can then deduce from this analysis the equations to predict the F-PEF (l/min) from the FEV1 (l/s) among males: F-PEF = 201 + 69.37 FEV1 and among females: F-PEF = 203 + 36.96 FEV1.

#### 3.2.6. Correlation between S-PEF and FEV1

Among males, there is a strong positive correlation between S-PEF and FEV1 (*r* = 0.68; *p* < 0.0001). Among females, there is also a positive correlation between S-PEF and FEV1 (*r* = 0.45; *p* < 0.0001). We can deduce from this analysis the predictive equations of F-PEF (l/min) from the FEV1 (l/s) among males: S-PEF= 165 + 96.70 FEV1 and among females: S-PEF = 193 + 61.34 FEV1.

Table 5 shows multiple correlation coefficients (*R*); determination coefficients (*R*^2^); Standard Error of Estimate (SEE); Residual Standard Deviation (RSD) for regression equations of F-PEF, S-PEF, and FEV1 in males and females.

## 4. Discussion

Our study is the first one in Morocco to establish predictive equations for PEF among adults between 18 and 70 years.

In addition to the richness of information we collected, the type of cross-sectional study has been privileged in our study considering its characters: being easy to design and make, quick, and inexpensive.

Our study took place in the city of Tetouan located in the north of Morocco, where altitude is very close to sea level (2 meters) eliminating any effects of living for years in high altitude on respiratory function [6]. Some studies took place in different cities with different altitudes [2, 3, 7–19].

Our study took place in a single city. This is the case for the majority of the studies [2, 7–9, 12–15, 17]. Studies in 2 different cities were rare [3, 18]. Nevertheless, a Japanese study investigated 5 different cities [19]; 2 Indian studies investigated 2000 people (50% women) living in a rural area of India [20] and 300 women in the Malawi region [11].

The equipment used, the standardization of methods and taking all measures by the same person, allowed a harmonized data collection avoiding any measurement variability, which ensured reliability of all of the results presented.

The spirometers used in the study met the criteria of ATS/ERS, which makes these measures and our results reliable and reproducible. The peak flow meter used is the Mini-Wright, known since 1977, which correlates well with the meter standard of Wright [4]. Compared to a spirometer, the resistance of this flow meter causes less variability in measurement but lowers the Peak Expiratory Flow [21]. This decline in the PEF between flow meter and spirometer has been proven in our study, with a greater decline in women (F-PEF = 21 + 0.78 S-PEF) than in men (F-PEF = 0.82 S-PEF). The volunteers included in the study had to be apparently healthy, that is, not having a current or earlier condition (acute or chronic). To do this, they had to answer the questionnaire (inclusion and exclusion criteria), and the physical examination has allowed us to select them. In retrospect, the exclusion of anyone with a pathological spirometry allowed us to form a good sample.

The criteria for inclusion and exclusion of our study were as strict as some studies [7, 20] and more stringent than those of other studies [2, 3, 9, 12, 18, 19]. For example, the Moroccan nationality of any volunteer and of both of his parents was required for inclusion in the study. Another example is the exclusion on ENT, osteoarthritis, neurological, and hematological affections, as well as pregnancy and obesity.

The size of the sample (*n* = 313) seems satisfactory. Our sample was more important than those of the Nigerian study of Bakki et al. (*n* = 255) [16], the Brazilian study of Paes et al. (*n* = 243) [7], the Indian studies of Vijayan et al. (*n* = 273) [13], Elebute and Femi-Pearse (*n* = 230) [17], and Dikshit et al. (*n* = 124) [10]; however it was less important than that of the Japanese study of Tsukioka et al. (*n* = 2785) [19]; Indian studies of Ray et al. (*n* = 2000) [20] and Prasad et al. (*n* = 897) [14]; the Palestinian study of Ghazal-Musmar et al. (*n* = 1000) [9]; the Indian study of Singh and Peri (*n* = 851) [12]; the Saudi study of Al-Taweel et al. (*n* = 680) [8]; English studies of Nunn and Gregg (*n* = 453) [3] (*n* = 401) [2]; and the Pakistani study of Hameed and Khan (*n* = 424) [18].

This can be explained by the fact that all our volunteers have underwent also a spirometry, a thing that was not done in the previous studies.

In our study, the sample included 143 men and 170 women; there is a slight predominance of women (54.3%). Ditto for Tsukioka et al. with 62.4% women [19]; for Nunn and Gregg with 55.76% [3]; for Ghazal-Musmar et al. with 51.8% [9]; and for Paes et al. with 50.61% [7]. In other studies we can note a masculine predominance as it is the case for Singh and Peri (53.11%) [12]; Hameed and Khan (50.23%) [18]; Gregg and Nunn (50.37%) [2]; Vijayan et al. (52.74%) [13]; Elebute and Femi-Pearse (61.7%) [17].

Only the Saudi study of Al-Taweel et al. included an exclusive sample of men [8]. Also, only the Indian study of Kaur was interested in an exclusive sample of women [11].

Our sample consisted of volunteers aged between 18 and 70 years. Only very advanced ages were not represented. Ranges of the other studies were from 15 to 84 years for Tsukioka et al. [19], 10 to 59 years for Ray et al. [20], 17 to 70 years for Singh and Peri [12], 14 to 64 years for Gregg and Nunn [2], 15 to 63 years for Vijayan et al. [13], 20 to 70 years for Paes et al. [7], and 10 to 60 years for Prasad et al. [14]. The average age of our study was 43.69 years. It is superior to those of Singh and Peri [12]; Hameed and Khan [18]; and Vijayan et al. [13] and this can be explained by the minimal age of their samples, which was, respectively, 17, 16 and, 15 years.

Our sample was made up of volunteers measuring between 137 and 192 cm. The average height was a ~166 cm. Outside the Brazilian sample [7] and Saudi sample [8], which had an average height greater than ours with 167.5 cm and 167.6 cm, respectively, other studies have found average heights less than our average, with 155.58 cm [20], 158.1 [12], and 159.3 [13] in Indian studies and 163.5 cm in the Pakistani study [18].

Our sample was made up of men and women weighing between 41 and 100 Kg. The average weight of men was of ~75 kg and that of women was of ~66 kg. Middleweight found in other studies was clearly lower at 2 Indian samples with 51.13 kg for Vijayan et al. [13] and 52.67 kg for Singh and Peri [12] and slightly lower in the Brazilian sample of Paes et al. with 68 kg.

The average BMI of our study was 25.29 kg/m^2^, and the study of Kaur et al. [11] has studied the BMI and found that the PEF is inversely proportional to the BMI.

In general, the gender and height, then age to lesser degree, and the weight very secondarily are considered as determinants of respiratory function [22]. These 4 parameters were influential for the F-PEF, the S-PEF, and FEV1 in our study. However, there are other physiological factors such as physical activity, temperature, altitude, ethnic differences, and environmental conditions that influence lung function [23–25].

It is established now that some respiratory parameters are higher in men than in women [25]. Similarly, there was a significant difference of the F-PEF, the S-PEF, and FEV1 in both sexes, most importantly in men. The slightly reduced values of women compared to men can be explained by physiological differences.

The most important objective of this study was to establish new equations for prediction of the flowmetric PEF. The prediction equations obtained in this study are used to calculate theoretical values of the PEF among Moroccan adults aged between 18 and 70 years.

A previous epidemiological study had attempted to define the optimal statistical model to represent the PEF. It studied 1239 normal subjects living in New Delhi, India. Different statistical models were tested to study the correlation between the PEF, on the one hand, and the age, weight and height on the other. It concluded that the linear model—used in our study—is simple and is the most accurate among the studied models [26].

The F-PEF among men and women in our study was correlated to age and height. This is consistent with several studies [2, 3, 8, 19, 20]. The average F-PEF in our sample was 366 l/min and in men it was 460 l/min and in women was 303 l/min.

Some studies have found higher values in men, with 524 l/min by Gregg and Nunn [2, 3]; 482 l/min by Elebute and Femi-Pearse [17]; 478 l/min by Hameed and Khan [18]; and 470 l/min by Malik et al. [27]; idem in women with 395 l/min by Kaur et al. [11]; 365 l/min with Elebute and Femi-Pearse [17]; 395 l/min by Gregg and Nunn [2, 3]; 422 l/min by Hameed and Khan [18]; and 323 l/min by Malik et al. [27].

The study of Vijayan et al. has found a correlation between the S-PEF and age, height, and weight [13]. This was the case of men in our sample, where the S-PEF was correlated with these 3 parameters. In women, the correlation was only with age and height.

For FEV1, the correlation found in both sexes was with age and height. Our study has objectified a good correlation between the FEV1, on one hand, and the F-PEF and S-PEF on the other hand (*p* < 0.05). Elebute and Femi-Pearse have found a good correlation between the FEV1 and PEF [17]. This will be very useful, since the measurement of the PEF by a flow meter in a structure not equipped with spirometer or anywhere outside health facilities may reflect the value of the FEV1 with good reliability.

Our study has several limitations, as the monocentric content of the study, the size of the sample, and the usage of a single brand of flow meter and spirometer. It is also important that other similar studies should be conducted in other parts of Morocco with similar or larger samples.

## 5. Conclusion

The reference values of the PEF in this study are original and useful standards to evaluate adults respiratory functions in Morocco. It is clear that, obtained by a flow meter, the PEF requires a correction coefficient. We can add that the flow meter can be considered as an alternative to the spirometer when the latter is not available, given the practical benefits it can bring.

## Figures and Tables

**Table 1:** The anthropometric and ventilatory characteristics of the sample.

Parameters	Sample (*N* = 313)		Men (*N* = 143)		Women (*N* = 170)		*p* value
	Mean ± SD	Range	Mean ± SD	Range	Mean ± SD	Range	
Age (year)	43.69 ± 13.35	18.06–70.33	43.69 ± 15.27	18.06–67.34	43.20 ± 12.59	18.28–70.33	0.21
Height (cm)	165.87 ± 7.69	137–192	174.08 ± 6.64	160–192	160.76 ± 7.43	137–180	0.34
Weight (Kg)	69.73 ± 10.54	41–100	75.33 ± 10.25	56–100	66.38 ± 9.2	41–91	0.09
BMI (m^2^/Kg)	25.29 ± 2.73	19.04–29.76	24.85 ± 2.98	19.04–29.76	25.61 ± 2.55	20.00–29.73	0.17
F-PEF (L/min)	366 ± 128	150–770	460 ± 127	190–770	303 ± 81	150–570	0.02
S-PEF (L/min)	426 ± 124	219–750	527 ± 113	253.8–750	360 ± 75	219–576	0.03
FEV1 (L/s)	3.14 ± 0.83	1.60–5.37	3.75 ± 0.44	2.56–5.37	2.73 ± 0.56	1.60–4.49	0.01

**Table 2:** The correlation of F-PEF with age, height, and weight in a simple and multiple linear regression among both sexes.

	Parameters	Correlation with	Correlation coefficient	*p* value
Men	*Pearson correlation*			
	Age	F-PEF	*r* = 0.317	*p* = 0.046
	Height		*r* = 0.354	*p* = 0.025
	Weight		*r* = 0.122	*p* = 0.454
	*Multiple linear regression*			
	Age	F-PEF	*r* = 0.194	*p* = 0.026
	Height		*r* = 0.263	*p* = 0.013

Women	* Pearson correlation*			
	Age	F-PEF	*r* = 0.782	*p* = 0.018
	Height		*r* = 0.641	*p* = 0.022
	Weight		*r* = 0.234	*p* = 0.08
	*Multiple linear regression*			
	Age	F-PEF	*r* = 0.317	*p* = 0.032
	Height		*r* = 0.307	*p* = 0.038

**Table 3:** The correlation of the S-PEF with age, height, and weight in simple and multiple linear regression among both sexes.

	Parameters	Correlation with	Correlation coefficient	*p* value
Men	* Pearson correlation*			
	Age	S-PEF	*r* = 0.306	*p* < 0.0001
	Height		*r* = 0.391	*p* < 0.0001
	Weight		*r* = 0.311	*p* < 0.0001
	*Multiple linear regression*			
	Age	S-PEF	*r* = 0.292	*p* < 0.0001
	Height		*r* = 0.172	*p* = 0.010
	Weight		*r* = 0.270	*p* < 0.0001

Women	*Pearson correlation*			
	Age	S-PEF	*r* = 0.224	*p* < 0.0001
	Height		*r* = 0.390	*p* < 0.0001
	Weight		*r* = 0.169	*p* = 0.004
	*Multiple linear regression (3 parameters)*			
	Age	S-PEF	*r* = 0.139	*p* = 0.024
	Height		*r* = 0.322	*p* < 0.0001
	Weight		*r* = 0.062	*p* = 0.338
	*Multiple linear regression (2 parameters)*			
	Age	S-PEF	*r* = 0.117	*p* = 0.041
	Height		*r* = 0.355	*p* < 0.0001

**Table 4:** The FEV1 correlation with age, height, and weight in simple and multiple linear regression among both sexes.

	Parameters	Correlation with	Correlation coefficient	*p* value
Men	*Pearson correlation*			
	Age	FEV1	*r* = 0.635	*p* < 0.0001
	Height		*r* = 0.596	*p* < 0.0001
	Weight		*r* = 0.204	*p* = 0.001
	*Multiple linear regression (3 parameters)*			
	Age	FEV1	*r* = 0.529	*p* < 0.0001
	Height		*r* = 0.393	*p* < 0.0001
	Weight		*r* = 0.089	*p* = 0.061
	*Multiple linear regression (2 parameters)*			
	Age	FEV1	*r* = 0.501	*p* < 0.0001
	Height		*r* = 0.444	*p* < 0.0001

Women	*Pearson correlation *			
	Age	FEV1	*r* = 0.610	*p* < 0.0001
	Height		*r* = 0.621	*p* < 0.0001
	weight		*r* = 0.137	*p* = 0.02
	*Multiple linear regression*			
	Age	FEV1	*r* = 0.479	*p* < 0.0001
	Height		*r* = 0.460	*p* < 0.0001
	Weight		*r* = 0.041	*p* = 0.372
	*Multiple linear regression (2 parameters)*			
	Age	FEV1	*r* = 0.465	*p* < 0.0001
	Height		*r* = 0.481	*p* < 0.0001

**Table 5:** Regression models for predicting F-PEF, S-PEF, and FEV1 in the Moroccan adults.

Sex	Variables	Regression parameters				*R*	*R* ^2^	SEE	RSD
		Constant	Age	Height	Weight				
Men	F-PEF	−374	−1.63	5.20	—	0.54	0.29	95	94
	S-PEF	−50	−2.11	2.61	2.75	0.64	0.41	89	87
	FEV1	−3.66	−0.03	0.05	—	0.76	0.58	0.51	0.51

Women	F-PEF	−235	−0.24	3.48	—	0.57	0.32	74	73
	S-PEF	−241	−0.61	3.79	—	0.66	0.43	65	65
	FEV1	−2.83	−0.02	0.04	—	0.76	0.58	0.35	0.35

*R*, multiple correlation coefficients; *R*^2^, determination coefficients; SEE, Standard Error of Estimate; RSD, Residual Standard Deviation.
